# The experiences of female surgeons around the world: a scoping review

**DOI:** 10.1186/s12960-020-00526-3

**Published:** 2020-10-28

**Authors:** Meredith D. Xepoleas, Naikhoba C. O. Munabi, Allyn Auslander, William P. Magee, Caroline A. Yao

**Affiliations:** 1 Division of Plastic and Maxillofacial Surgery, Children’s Hospital Los Angeles, Los Angeles, CA USA; 2 Operation Smile Inc, Virginia Beach, Virginia Beach, VA USA; 3 Division of Plastic and Reconstructive Surgery, Keck School of Medicine, 1510 San Pablo St, Suite 415, Los Angeles, CA USA; 4 Division of Plastic Surgery, Shriners Hospital for Children, Los Angeles, CA USA

**Keywords:** Female surgeon, Gender equity, Women in surgery, Surgeons, Female, Surgical  workforce, Global surgery

## Abstract

**Introduction:**

The Lancet Commission for Global Surgery identified an adequate surgical workforce as one indicator of surgical care accessibility. Many countries where women in surgery are underrepresented struggle to meet the recommended 20 surgeons per 100,000 population. We evaluated female surgeons’ experiences globally to identify strategies to increase surgical capacity through women.

**Methods:**

Three database searches identified original studies examining female surgeon experiences. Countries were grouped using the World Bank income level and Global Gender Gap Index (GGGI).

**Results:**

Of 12,914 studies meeting search criteria, 139 studies were included and examined populations from 26 countries. Of the accepted studies, 132 (95%) included populations from high-income countries (HICs) and 125 (90%) exclusively examined populations from the upper 50% of GGGI ranked countries. Country income and GGGI ranking did not independently predict gender equity in surgery. Female surgeons in low GGGI HIC (Japan) were limited by familial support, while those in low income, but high GGGI countries (Rwanda) were constrained by cultural attitudes about female education. Across all populations, lack of mentorship was seen as a career barrier. HIC studies demonstrate that establishing a critical mass of women in surgery encourages female students to enter surgery. In HICs, trainee abilities are reported as equal between genders. Yet, HIC women experience discrimination from male co-workers, strain from pregnancy and childcare commitments, and may suffer more negative health consequences. Female surgeon abilities were seen as inferior in lower income countries, but more child rearing support led to fewer women delaying childbearing during training compared to North Americans and Europeans.

**Conclusion:**

The relationship between country income and GGGI is complex and neither independently predict gender equity. Cultural norms between geographic regions influence the variability of female surgeons’ experiences. More research is needed in lower income and low GGGI ranked countries to understand female surgeons’ experiences and promote gender equity in increasing the number of surgical providers.

## Introduction

In the modern era of medicine, Elizabeth Blackwell was the first reported woman to graduate from medical school in 1849 and pursue a career in surgery [1]. Women pursuing careers in medicine has steadily increased with women now representing 50% of current medical school matriculants in the United States (US) [2]. This shift is not reflected to the same extent in surgical specialties, where women have experienced much slower growth [1]. In the United Kingdom (UK) and the US, men are 73% and 61.6% of practicing surgeons, respectively [3, 4]. The number of female surgeons in low- and middle-income countries rose disproportionately slower than female representation in other medical specialties [5–7]. Concurrently, five-billion people lack access to safe, affordable surgical care globally and many countries need an increase in surgical providers to reach the recommended 20 per 100,000 population [6]. With the majority of low- and middle-income countries struggling to build an adequate surgical workforce, expanding the participation of women in surgery is a powerful way to help alleviate the global burden of surgery [6, 7].

The experiences of women in medicine and how they differ from men is well documented. The majority of this work has focused on barriers such as discrimination, pay gaps, and promotion inequality [8–11]. Surgery continues to be a male-dominated field with the disparate experiences between genders not well documented worldwide. Understanding career experiences of women in surgery is essential to expand the female workforce, improve the professional surgical environment, and retain existing female surgeons.

This scoping review seeks to understand the experiences of female surgeons around the world and how they differ based on geography, national income (World Bank income level) and cultural beliefs of gender equity (Global Gender Gap Index (GGGI)). The experience of female surgeons is a very broad topic for which we hope to synthesize the current knowledge and identify where gaps in gender equity are evident globally. Our analysis can inform future training programs and professional, educational and institutional initiatives and policies. We hope to inspire new strategies to increase surgical capacity through empowering women globally.

## Methods

A scoping review was conducted following the Preferred Reporting Items for Systematic Reviews and Meta Analyses extension for Scoping Reviews (PRISMA-ScR) [12] guidelines for reporting (Additional file 1). A detailed protocol has been provided as Additional file 2.

### Research question

This review was led by the question, ‘What are the experiences of female surgeons around the world and how to do they differ based on geography, country income level, and cultural beliefs of gender equity?’ The female surgical experience was defined as any difference in attitude, treatment, behavior or career outcome that results from a surgeon’s female gender.

### Inclusion/exclusion criteria

Included were original, peer-reviewed, full-text articles published in English that studied female surgeons, female surgical residents, and female medical students considering surgery. Topics required for inclusion were work–life balance, salary, health, job titles, career factors and barriers, training, skills, pregnancy, childrearing, domestic work, volunteerism, interpersonal interactions and discrimination/harassment. All study types were included, such as cross-sectional analysis, questionnaires, longitudinal analysis, and controlled trials. Editorials, case reports and personal anecdotes were excluded due to potential bias. No restriction was placed on the year of publication to assess the complete literature on female surgeons.

### Search strategy, study selection and data collection

A search of PubMed, Web of Science, and MEDLINE (Ovid) was conducted on April 2, 2020 and included six search constructs (Table 1). One author (M.X.) conducted the initial review and excluded articles that did not meet inclusion criteria according to title. Two authors (M.X. and N.M.) reviewed the remaining study abstracts and excluded articles that did not meet inclusion criteria. The remaining articles were summarized in a chart in Microsoft Excel 2013 (Microsoft Corporation, Redmond, WA). Full-text articles were individually reviewed by two authors (M.X. and N.M.) to extract study characteristics including study design, publication year, study population countries and gender distribution, the category of the female surgical experience, funding source, and the study’s main findings. Studies that did not meet the inclusion criteria were excluded. Any inclusion discrepancies between authors was resolved through discussion. Data from included studies was compiled into a single spreadsheet for analysis independently.

**Table 1:** Search terms and results from each database

Included search terms	Results from PubMed	Results from web of science	Results from MEDLINE (Ovid)
“Female Surgeons”	201	46	329
“Women Surgeons”	130	124	257
Women in Surgery [Title]*	58	53	236
Female Surgeon [Title]*	9	6	91
Female “Surgical Training”*	1299	252	2711
Female “Surgical Experience”*	2165	252	4695
Totals	3862	733	8319
Total results	12,914		

* Search terms configured after the primary search to keep search results relevant to the study questions

### Synthesis of results

Studies were sorted into four key categories based on main focus: careers challenges, residency and training, family and work–life balance, and other. The World Bank Income Level Group and GGGI ranking of included countries were recorded. The World Bank classifies countries into four categories according to gross national income per capita: low-income country (LIC), lower-middle income country (LMIC), upper-middle income country (UMIC), and high-income country (HIC) [13]. These income-level groupings indicate a country’s economic capabilities, associated resources, and opportunities that may be available to the population within. The Global Gender Gap Index is a weighted rating comprising of scores for economic participation and opportunity, educational attainment, health and survival, and political empowerment. GGGI ratings contextualize the experiences of women around the world in a social and professional capacity. Lower scores and rankings correspond to less equality for women [14]. Summary and descriptive statistics were calculated using Microsoft Excel 2013.

## Results

**Fig. 1:**
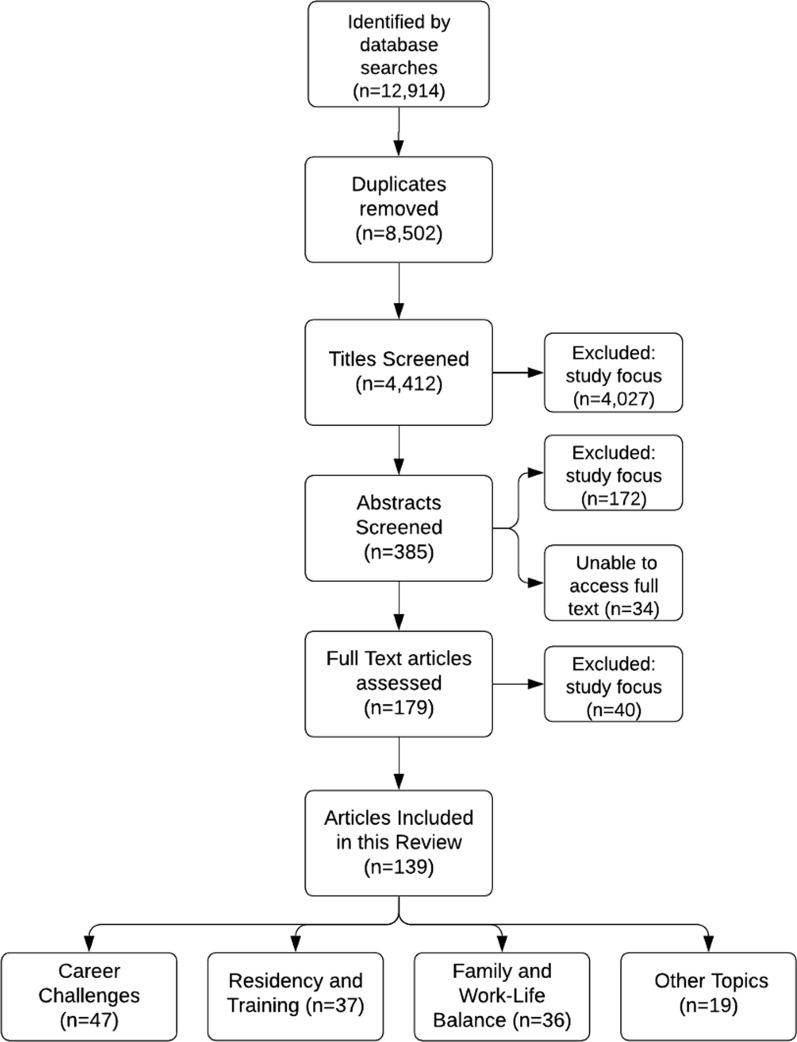
The methods of screening articles for this review

The PubMed search yielded 12,914 total articles. A total of 12,775 articles were excluded as duplicates, having incorrect study focus, or not being original studies published in peer-reviewed journals (Fig. 1). The process yielded 139 studies meeting inclusion criteria and published between 1993 and 2020 (Fig. 1, Table 2). Of these 139 articles, 66% (*n* = 92) were published in 2015 or later (Table 2). Of the included articles, 47 (34%) focused on careers challenges, 37 (27%) on residency and training, 36 (26%) on family and work–life balance, and 19 (14%) on other topics (Fig. 1). The category of “other” included articles related to interpersonal interactions (*n* = 3), salary (*n* = 8), physical health (*n* = 5), demographics (*n* = 2), and international volunteerism (*n* = 1). Included study details appear in Table 2. The most common methodology of the articles was questionnaire (*n* = 77, 55.0%), cross-sectional (*n* = 23, 16.4%), and semi-structured or qualitative interview (*n* = 10, 7.4%).

**Table 2:** Full list of articles included in review organized according to topic category

	Career challenges						
Year	Title	World Bank income group	Country	Study design	Population size	Gender distribution (F/M) % Female	Funding source
2020	A Call to Action: Black/African American Women Surgeon Scientists, Where are They? [87]	High income	United States	Retrospective review	*n* = 123		Not reported
2020	A Report on the Representation of Women in Academic Plastic Surgery Leadership. [88]	High income	United States	Retrospective review			Not reported
2020	Gender and academic promotion of Canadian general surgeons: a cross-sectional study. [89]	High income	Canada	Cross-sectional analysis	*n* = 405	(111/294) 27%	Not reported
2020	Gender Disparities Among Burn Surgery Leadership. [90]	High income	United States	Cross-sectional analysis	*n* = 581	(58/523) 10%	No funding
2020	Gender Disparity Among Surgical Peer-Reviewed Literature. [91]	High income	United States	Retrospective review			Not reported
2020	Influence of gender on career expectations of oral and maxillofacial surgeons.[92]	Lower middle, upper middle and high income	Egypt Jordan Saudi Arabia	Questionnaire	*n* = 110	(40/70) 36%	Not reported
2020	Perceptions on gender disparity in surgery and surgical leadership: A multicenter mixed methods study. [93]	High income	United states	Mixed methods	*n* = 36	(14/22) 39%	No funding
2020	Gender disparities in academic vascular surgeons. [94]	High income	United States	Cross-sectional analysis	*n* = 951	(117/774) 19%	Not reported
2020	Gender disparity and sexual harassment in vascular surgery practices. [95]	High income	United States	Questionnaire	*n* = 149	(33/116) 22%	Not reported
2019	Barriers to careers identified by women in academic surgery: A grounded theory model. [21]	High income	United States	Semi-structured interviews	*n* = 15	100%	No funding
2019	Female Representation and Implicit Gender Bias at the 2017 American Society of Colon and Rectal Surgeons' Annual Scientific and Tripartite Meeting.[96]	High income	United States	Prospective observational study	*n* = 1532	100%	No funding
2019	Gender differences among surgical fellowship program directors.[97]	High income	United States	Cross-sectional analysis	*n* = 811		Not reported
2019	Is Gender Associated With Success in Academic Oral and Maxillofacial Surgery?[98]	High income	United States	Cross-sectional analysis	*n* = 306	(53/253) 17%	Not reported
2019	Military Medicine and the Academic Surgery Gender Gap. [99]	High income	United States	Cross-sectional analysis	*n* = 2125	(376/1749) 18%	Not reported
2019	Assessment of Gender Differences in Perceptions of Work–Life Integration Among Head and Neck Surgeons. [100]	High income	United States	Questionnaire	*n* = 261	(71/190) 27%	Not reported
2019	A woman's place is in theatre: women's perceptions and experiences of working in surgery from the Association of Surgeons of Great Britain and Ireland women in surgery working group. [24]	High income	United Kingdom Ireland	Questionnaire	*n* = 81	100%	Not-for-profit sponsored
2019	Despite Growing Number of Women Surgeons, Authorship Gender Disparity in Orthopaedic Literature Persists Over 30 Years. [101]	High income	United States	Cross-sectional analysis	*n* = 6		Not reported
2019	Editorial (Spring) Board? Gender Composition in High-impact General Surgery Journals Over 20 Years. [102]	High income	United States	Cross-sectional analysis	*n* = 10		Public-Sponsored
2019	Gender Disparity in Surgery: An Evaluation of Surgical Societies. [103]	High income	United States	Cross-sectional analysis	*n* = 587	(135/452) 23%	Not reported
2019	Gender representation in leadership roles in UK surgical societies. [3]	High income	United Kingdom	Cross-sectional analysis	*n* = 20,803	(2446/18,357) 12%	No funding
2019	Is there a gender bias in the advancement to SAGES leadership? [104]	High income	United States	Retrospective longitudinal analysis	*n* = 1546	(323/1223) 21%	Not reported
2019	Change Is Happening: An Evaluation of Gender Disparities in Academic Plastic Surgery. [105]	High income	United States	Cross-sectional analysis	*n* = 938	(186/752) 20%	No funding
2019	Gender disparities in academic rank achievement in neurosurgery: a critical assessment. [106]	High income	United States	Cross-sectional analysis	*n* = 841	(80/761) 10%	Not reported
2019	Gender Disparity in Leadership Positions of General Surgical Societies in North America, Europe, and Oceania. [20]	High income	United States Australia New Zealand (Europe)^Δ^	Retrospective cross-sectional analysis			Not reported
2019	Practice patterns and work environments that influence gender inequality among academic surgeons. [107]	High income	United States	Retrospective cross-sectional analysis	*n* = 51	(10/41) 20%	No funding
2019	Female Neurosurgeons in Europe-On a Prevailing Glass Ceiling. [16]	Lower middle Upper middle and High income	22 Countries^†^	Questionnaire	*n* = 116	100%	No funding
2018	Female Surgeons as Counter Stereotype: The Impact of Gender Perceptions on Trainee Evaluations of Physician Faculty. [108]	High income	United States	Cross-sectional analysis	*n* = 1066	(467/599) 44%	Not reported
2018	Organizational barriers to and facilitators for female surgeons' career progression: a systematic review. [109]	High income	United Kingdom United States Canada	Systematic review			No funding
2017	Discrimination against female surgeons is still alive: Where are the full professorships and chairs of departments? [110]	High income	United States	Systematic review		100%	Not reported
2017	E-WIN Project 2016: Evaluating the Current Gender Situation in Neurosurgery Across Europe-An Interactive, Multiple-Level Survey [17]	Lower middle Upper middle and High income	35 countries*	Questionnaire	*n* = 12,985	12%	Not reported
2017	Gender Differences in the Professional and Personal Lives of Plastic Surgeons [111]	High income	United States	Questionnaire	*n* = 757	(309/448) 41%	Not reported
2016	Gender Differences in Pediatric Orthopedics: What Are the Implications for the Future Workforce? [112]	High income	United States	Questionnaire	*n* = 62	(18/44) 29%	Not reported
2016	The erasure of gender in academic surgery: a qualitative study. [113]	High income	Canada	Qualitative interviews	*n* = 8	100%	Not reported
2015	Surgeons in Difficulty: An Exploration of Differences in Assistance-Seeking Behaviors between Male and Female Surgeons. [114]	High income	United States	Questionnaire	*n* = 192	(113/79) 59%	Not reported
2015	Women in surgery: factors deterring women from being surgeons in Zimbabwe. [27]	Lower middle income	Zimbabwe	Questionnaire	*n* = 159	(74/85) 46%	Not reported
2014	Gender inequality in career advancement for females in Japanese academic surgery. [115]	High income	Japan	Quantitative/evaluation study	*n* = 787	(132/655) 17%	Not reported
2013	Perceived gender-based barriers to careers in academic surgery. [116]	High income	United States	Questionnaire	*n* = 154	(70/84) 46%	Not reported
2011	Is there still a glass ceiling for women in academic surgery? [22]	High income	United States	Scoping review		100%	Not reported
2011	Under representation of women in surgery in Nigeria: by choice or by design?[25]	Lower middle income	Nigeria	Questionnaire	*n* = 105	100%	Not reported
2010	Women in surgery: a survey in Switzerland. [23]	High income	Switzerland	Questionnaire	*n* = 189	100%	No funding
2009	Practice patterns and career satisfaction of Canadian female general surgeons [117]	High income	Canada	Questionnaire	*n* = 85	100%	Not reported
2006	Challenges confronting female surgical leaders: Overcoming the barriers [26]	High income	United States	Semi-structure interviews	*n* = 10	100%	Not reported
2004	Women in academic general surgery.[118]	High income	United States	Questionnaire	*n* = 317	(149/168) 47%	Not Reported
2004	Professional satisfaction of women in surgery: results of a national study. [119]	High income	Austria	Questionnaire	*n* = 206	100%	Not-for-profit-sponsored
2001	Collective contributions of women to cardiothoracic surgery: a perspective review. [120]	High income	United States	Cross-sectional analysis	*n* = 84	100%	Not reported
2000	Perceived obstacles to career success for women in academic surgery. [121]	High income	United States	Questionnaire/systematic review	*n* = 54	(9/45) 17%	Not reported
1996	Women in oral and maxillofacial surgery: factors affecting career choices, attitudes, and practice characteristics. [122]	High income	United States	Questionnaire	*n* = 107	100%	Not reported

	Residency and training						
Year	Title	World Bank Income Group	Country	Study design	Population size	Gender distribution (M/F) % Female	Funding source
2020	Barriers to Women Entering Surgical Careers: A Global Study into Medical Student Perceptions. [18]	All levels	75 Countries^*ψ*^	Questionnaire	*n* = 639	(374/265) 59%	Not reported
2020	Sexual Harassment and Cardiothoracic Surgery: #UsToo? [15]	Unknown	Unknown^∓^	Questionnaire	*n* = 790	(185/591) 23%	Not reported
2020	Women Continue to Be Underrepresented in Surgery: A Study of AMA and ACGME Data from 2000 to 2016. [123]	High income	United States	Retrospective Review			Not Reported
2020	Women in otolaryngology in Turkey: Insight of gender equality, career development and work–life balance. [29]	Upper middle income	Turkey	Questionnaire	*n* = 156	100%	No funding
2019	Paradox of meritocracy in surgical selection, and of variation in the attractiveness of individual specialties: to what extent are women still disadvantaged?[124]	High income	Australia New Zealand	Cross-Sectional Analysis	*n* = 5288		Not reported
2019	Gender Differences in Case Volume Among Ophthalmology Residents. [125]	High income	United States	Retrospective longitudinal analysis	*n* = 1271	(456/815) 36%	Private-sponsored
2019	Understanding the Barriers to Reporting Sexual Harassment in Surgical Training. [41]	High income	United States	Questionnaire	*n* = 270	(120/143) 44%	Not reported
2019	Why do women leave surgical training? A qualitative and feminist study. [46]	High income	Australia New Zealand	Qualitative interviews	*n* = 12	100%	Not-for-profit-sponsored
2019	Assessing gender bias in qualitative evaluations of surgical residents. [126]	High income	United States	Qualitative analysis	*n* = 143	(51/92) 36%	Public sponsored
2019	Female Medical Student Retention in Neurosurgery: A Multifaceted Approach. [51]	High income	United States	Questionnaire	*n* = 104	100%	Not reported
2019	Gender Bias Experiences of Female Surgical Trainees. [43]	High income	United States	Mixed methods	*n* = 48	100%	University-sponsored
2018	A qualitative study on perceptions of surgical careers in Rwanda: A gender-based approach. [30]	Low income	Rwanda	Semi-structured interviews	*n* = 12	(6/6) 50%	No funding
2018	A qualitative study of gender differences in the experiences of general surgery trainees. [36]	High income	United States	Structured interviews	*n* = 42	(18/24) 43%	No Funding
2018	Burnout and gender in surgical training: A call to re-evaluate coping and dysfunction. [127]	High income	United States	National survey	*n* = 566	(288/278) 51%	No funding
2017	Does gender impact on female doctors ‘experiences in the training and practice of surgery? A single center study. [28]	Upper middle income	South Africa	Questionnaire	*n* = 32	100%	Not reported
2016	Understanding and Overcoming Implicit Gender Bias in Plastic Surgery. [35]	High income	United States	Systematic review			Not reported
2016	A Values Affirmation Intervention to Improve Female Residents' Surgical Performance. [49]	High income	United States	Randomized control trial	*n* = 93	(32/61) 35%	University-sponsored
2016	Exploring the Relationship Between Stereotype Perception and Residents' Well-Being. [37]	High income	United States	Correlation study	*n* = 384	(189/195) 49%	University-sponsored
2016	Medical School Experiences Shape Women Students' Interest in Orthopaedic Surgery. [38]	High income	United States United Kingdom	Systematic Review			Not reported
2016	Mentorship as Experienced by Women Surgeons in Japan. [52]	High income	Japan	Questionnaire	*n* = 55	100%	Not reported
2016	Women in academic surgery: why is the playing field still not level? [34]	High income	Canada	Questionnaire	*n* = 81	100%	No funding
2015	Gender differences in the acquisition of surgical skills: a systematic review. [48]	High income	United States United Kingdom Sweden Canada Denmark Switzerland	Systematic review	*n* = 2106		Not reported
2015	Perceptions of gender-based discrimination during surgical training and practice. [39]	High income	United States	Questionnaire	*n* = 334	100%	No funding
2013	Women in surgical residency training programs. [5]	High income	United States	Cross-Sectional Analysis			Not reported
2013	Gender-related perceptions of careers in surgery among new medical graduates: results of a cross-sectional study. [128]	High income	United Kingdom	Questionnaire	*n* = 208	(130/78) 63%	Not reported
2013	The only girl in the room: how paradigmatic trajectories deter female students from surgical careers [45]	High income	United Kingdom	Semi-structure interviews	*n* = 19	60%	University-sponsored
2011	Differences in final product of a bowel anastomosis of male and female novice surgeons. [47]	high income	United Kingdom	Non-randomized control trial	*n* = 36	(18/18) 50%	Not reported
2011	Women in surgery residency programs: evolving trends from a national perspective. [129]	High income	United States	Cross-sectional analysis			Not reported
2009	Burnout in Australasian Younger Fellows. [130]	High income	Australia	Questionnaire	*n* = 277	(52/225) 19%	Not-for-profit-sponsored
2009	Sex and the orthopaedic surgeon: a survey of patient, medical student and male orthopaedic surgeon attitudes towards female orthopaedic surgeons. [44]	High income	United Kingdom	Questionnaire	*n* = 561	(284/277) 51%	Not reported
2006	Women in surgery: do we really understand the deterrents? [42]	High income	United States	Questionnaire	*n* = 141	(90/51) 64%	Not reported
2005	The training needs and priorities of male and female surgeons and their trainees. [40]	High income	United States	Questionnaire	n = 4308	(1034/3274) 24%	Not reported
2005	Why are women deterred from general surgery training? [33]	High income	Canada	Questionnaire	*n* = 417	(314/103) 75%	Not-for-profit-sponsored
2002	Perceptions of women medical students and their influence on career choice. [50]	High income	United States	Questionnaire	*n* = 305	100%	Not reported
2000	The influence of gender and specialty on reporting of abusive and discriminatory behaviour by medical students, residents and physician teachers.[31]	High income	Canada	Questionnaire	*n* = 569	(212/357) 38%	Not reported
2000	A surgical career? The views of junior women doctors. [131]	High income	United Kingdom	Structured interviews	*n* = 24	(12/12) 50%	Not reported
1996	Do Canadian female surgeons feel discriminated against as women? [32]	High income	Canada	Questionnaire	*n* = 419	100%	Not-for-profit-sponsored

	Family and work–life balance						
Year	Title	World Bank Income Group	Country	Study design	Population size	Gender distribution (M/F) % Female	Funding source
2020	Surgeon Experience with Parental Leave Policies Varies Based on Practice Setting. [132]	High income	United States	Questionnaire	*n* = 477	100%	No funding
2019	Surgical trainees' experience of pregnancy, maternity and paternity leave: a cross-sectional study. [61]	High income	United Kingdom	Questionnaire	*n* = 876	(555/321) 63%	No funding
2019	An Analysis of Differences in the Number of Children for Female and Male Plastic Surgeons [59]	High income	United States	Questionnaire	*n* = 757	309/448 41%	No funding
2019	Policies and practice regarding pregnancy and maternity leave: An international survey. [19]	Lower middle Upper middle High income	United States United Kingdom Canada Nigeria Australia China Sweden Ireland Israel Finland Italy South Africa^°^	Questionnaire	*n* = 1111	100%	No funding
2018	Factors Associated With Residency and Career Dissatisfaction in Childbearing Surgical Residents. [55]	High income	United States	Questionnaire	*n* = 347	100%	Not reported
2018	Pregnancy and Motherhood During Surgical Training. [69]	High income	United States	Questionnaire	*n* = 342	100%	Not reported
2018	Women in surgery: A longer term follow-up. [133]	High income	United States	Cross-Sectional Analysis	*n* = 108	(26/82) 24%	Not-for-profited sponsored
2018	Female trainees believe that having children will negatively impact their careers: results of a quantitative survey of trainees at an academic medical center [67]	High income	United States	Questionnaire	*n* = 435	261/174 60%	University-sponsored
2018	Perspectives of pregnancy and motherhood among general surgery residents: A qualitative analysis [134]	High income	United States	Questionnaire	*n* = 219	100%	Not reported
2017	WOMEN IN SURGERY—an overview of the evolving trends in Nigeria. [53]	Lower middle income	Nigeria	Questionnaire	*n* = 60	100%	Not reported
2017	Gender differences in academic surgery, work–life balance, and satisfaction. [73]	High income	United States	Questionnaire	*n* = 243	(76/167) 31.3%	Not reported
2017	Career intentions of female surgeons in German liver transplant centers considering family and lifestyle priorities. [135]	High income	Germany	Questionnaire	*n* = 81	100%	Not reported
2016	Factors that Can Promote or Impede the Advancement of Women as Leaders in Surgery: Results from an International Survey. [76]	High income	United States Japan Finland Hong Kong (SAR, China)	Questionnaire	*n* = 225	100%	Not reported
2016	Suturing the gender gap: Income, marriage, and parenthood among Japanese Surgeons. [136]	High income	Japan	Questionnaire	*n* = 1938	(846/1092) 43.7%	Not reported
2016	Biographic Characteristics and Factors Perceived as Affecting Female and Male Careers in Academic Surgery: The Tenured Gender Battle to Make It to the Top. [70]	High income	Germany	Questionnaire	*n* = 133	(63/70) 47.4%	No funding
2016	Why Do Women Choose to Enter Academic Oral and Maxillofacial Surgery? [58]	High income	United States	Questionnaire	*n* = 31	100%	Not reported
2016	Working Conditions and Lifestyle of Female Surgeons Affiliated to the Japan Neurosurgical Society: Findings of Individual and Institutional Surveys. [63]	High income	Japan	Questionnaire	*n* = 224	100%	Not reported
2015	'You become a man in a man's world': is there discursive space for women in surgery? [137]	High income	United Kingdom	Semi-structured interviews	*n* = 15	100%	No funding
2014	Does a surgical career affect a woman's childbearing and fertility? A report on pregnancy and fertility trends among female surgeons. [68]	High income	United States	Questionnaire	*n* = 1021	100%	Not reported
2014	Pregnancy-Related Attrition in General Surgery [60]	High income	United States	Retrospective Review	*n* = 85	(36/49) 42%	Not reported
2014	Work–life balance of female versus male surgeons in Hong Kong based on findings of a questionnaire designed by a Japanese surgeon. [71]	High income	Hong Kong (SAR, China)	Questionnaire	*n* = 114	(37/77) 32.5%	Not reported
2012	Childbearing and pregnancy characteristics of female orthopaedic surgeons. [66]	High income	United States	Questionnaire	*n* = 1021	100%	No funding
2012	Pregnancy among women surgeons: trends over time. [56]	High income	United States	Questionnaire	*n* = 1950	100%	Not-for-profit-sponsored
2011	Relationship between work–home conflicts and burnout among American surgeons: a comparison by sex. [65]	High income	United States	Questionnaire	*n* = 7858	(1043/6815) 13%	Not-for-profit-sponsored
2011	Female surgeons' mentoring experiences and success in an academic career in Switzerland. [75]	High income	Switzerland	National Survey	*n* = 189	100%	Private-sponsored
2010	Women surgeons in Hong Kong [138]	High income	Hong Kong (SAR, China)	Questionnaire	*n* = 172	(58/114) 34%	Not reported
2010	Career satisfaction of women in surgery: perceptions, factors, and strategies. [139]	High income	United States	Semi-structured interviews	*n* = 18	(12/6) 66.7%	Not reported
2009	Women surgeons in the new millennium. [57]	High income	United States	Questionnaire	*n* = 895	(178/698) 20%	Not-For-profit-sponsored
2009	Gender and Specialty Influences on Personal and Professional Life Among Trainees. [140]	High income	Ireland	Questionnaire	*n* = 460	(300/160) 65%	Not reported
2004	The gender gap in a surgical subspecialty—Analysis of career and lifestyle factors [141]	High income	United States	Questionnaire	*n* = 673	37%	Not-for-profit-sponsored
2003	Career satisfaction and surgical practice patterns among female ophthalmologists [74]	High income	Canada	Questionnaire	*n* = 137	72/65 45%	Not reported
2001	Childbearing and childcare in surgery. [54]	High income	United States	Questionnaire	*n* = 42	(20/22) 47.6%	Not reported
1998	Characteristics of women surgeons in the United States. [142]	High income	United States	Questionnaire	*n* = 4445	100%	Not reported
1997	Plastic surgeons: a gender comparison. [72]	High income	United States	Questionnaire	*n* = 432	(216/216) 50%	Not reported
1994	Women surgeons: career and lifestyle comparisons among surgical subspecialties. [64]	High income	Canada	Questionnaire	*n* = 419	100%	Not-for-profit-sponsored
1993	Women surgeons. Results of the Canadian Population Study. [62]	High income	Canada	Questionnaire	*n* = 419	100%	Not reported

	Other (pay, etc.)						
Year	Title	World Bank Income Group	Country	Study design	Population size	Gender distribution (F/M)% Female	Funding source
2020	Gender Disparity in Trauma Surgery: Compensation, Practice Patterns, Personal Life, and Wellness. [143]	High income	United States	Questionnaire	*n* = 497	105/356 21%	Not reported
2020	Gender and compensation among surgical specialties in the Veterans Health Administration. [144]	High income	United States	Cross-sectional analysis	*n* = 1993	458/1535 23%	Not reported
2020	The Role of Gender, Academic Affiliation, and Subspecialty in Relation to Industry Payments to Orthopaedic Surgeons [145]	High income	United States	Retrospective Analysis	*n* = 22,352	1299/21,053 6%	No funding
2020	Men Receive Three Times More Industry Payments than Women Academic Orthopaedic Surgeons, Even After Controlling for Confounding Variables. [146]	High income	United States	Retrospective cross-sectional analysis	*n* = 2893	316/2577 11%	Not reported
2019	Gender Equity in Humanitarian Surgical Outreach: A Decade of Volunteer Surgeons.[147]	High income	United States	Questionnaire	*n* = 227	(139/88) 61%	Not reported
2019	Women surgeons and the emergence of acute care surgery programs. [148]	High income	United States	National survey	*n* = 1546		Public-sponsored
2019	Sex-Based Disparities in the Hourly Earnings of Surgeons in the Fee-for-Service System in Ontario, Canada.[149]	High income	Canada	Cross-sectional analysis	*n* = 3275		Private-sponsored
2019	The Effect of Sex on Orthopaedic Surgeon Income. [81]	High income	United States	Questionnaire	*n* = 4335	302/4033 7%	No funding
2018	The surgical personality: does it exist? [150]	High income	United Kingdom	Questionnaire	*n* = 599	(256/341) 43%	Not reported
2018	The ties that bind: what's in a title? [151]	High income	Australia New Zealand	Retrospective review	*n* = 6143	(702/5441) 11.4%	Not reported
2018	A Structured Compensation Plan Improves But Does Not Erase the Sex Pay Gap in Surgery. [152]	High income	United States	Questionnaire	*n* = 44	(11/33) 25%	Not reported
2018	Assessing the domino effect: Female physician industry payments fall short, parallel gender inequalities in medicine. [153]	High income	United States	Cross-sectional analysis	*n* = 31,297	(4511/26,786) 14%	No funding
2018	Equal Pay for Equal Work: Medicare Procedure Volume and Reimbursement for Male and Female Surgeons Performing Total Knee and Total Hip Arthroplasty. [154]	High income	United States	Cross-sectional analysis	*n* = 20,546	(906/19,640) 4.4%	No funding
2018	Can the surgeon live his whole life? Analysis of the risk of death related to the profession. [77]	High income	Poland	Cross-sectional analysis	*n* = 6496		Not reported
2015	The Nonwhite Woman Surgeon: A Rare Species. [155]	High income	United States	Questionnaire	*n* = 194	(81/113) 42%	Not reported
2015	Cancer Prevalence among a Cross-sectional Survey of Female Orthopedic, Urology, and Plastic Surgeons in the United States. [79]	High income	United States	National survey	*n* = 1023	100%	Not reported
2014	The ergonomics of women in surgery.[80]	High income	United States	Questionnaire	*n* = 314	(54/260) 17.2%	Not reported
2014	Perceptions of surgeons: what characteristics do women surgeons prefer in a colleague? [156]	High income	United States	Questionnaire	*n* = 212	100%	University-sponsored
2005	Female surgeons' alcohol use: a study of a national sample of Norwegian doctors. [78]	High income	Norway	Questionnaire	*n* = 1385	(347/1038) 25%	Not reported

Blank boxes indicate that data could not be found or did not apply

* Did not analyze data according to the 35 Countries in this study: Albania, Austria, Belgium, Bosnia-Herzegovina, Bulgaria, Croatia, Cyprus, Czech Republic, Denmark, Estonia, Finland, France, Germany, Greece, Hungary, Israel, Italy, Kazakhstan, Kosovo, Latvia, Lithuania, Moldova, Netherlands, Poland, Portugal, Romania, Russia, Serbia, Slovakia, Spain, Sweden, Switzerland, Turkey, Ukraine, and United Kingdom

^Δ^Did not analyze data according to country

^†^Did not analyze data according to the 22 Countries in this study: Austria, Belgium, Croatia, Czech Republic, Estonia, France, Germany, Greece, Ireland, Italy, Latvia, Netherlands, Poland, Portugal, Russia, Serbia, Spain, Sweden, Switzerland, Turkey, Ukraine, and United Kingdom

^*ψ*^Included countries were indicated in a map in the article, therefore reliable data on exact countries could not be completely determined

^∓^Only these 3 countries were listed: United States, Canada and Mexico. The rest of the study population was designated by continent only. A complete list could not be determined for this study as results did not analyze differences between countries

^°^Respondents from 53 countries participated in this study, but the authors only reported countries with > 10 responses in their paper

### Geography, World Bank income level and GGGI

**Fig. 2:**
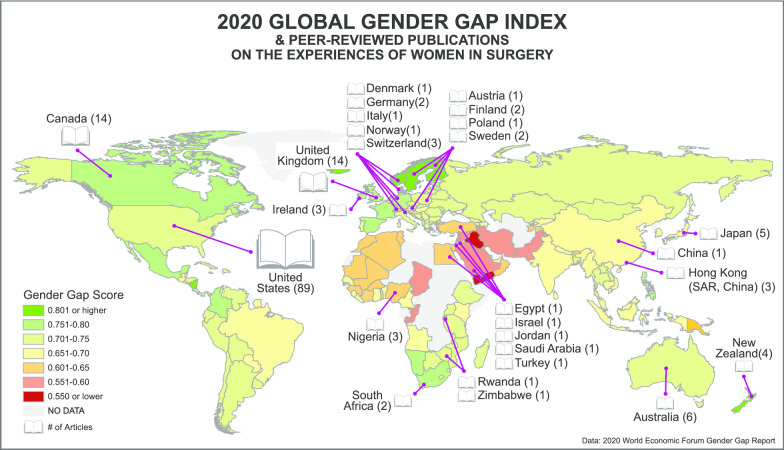
The number of studies per country overlaid on a 2020 heat map of the Global Gender Inequality Index

Fifteen studies examined populations from multiple countries (Table 2). Most study populations originated from the North America (*n* = 103, 62.4%) and Europe (*n* = 31, 18.8%). Remaining study populations originated from Asia (*n* = 13, 7.9%), Oceania (*n* = 10, 6.1%), and Africa (*n* = 8, 4.8%) (Table 3). No studies evaluated female surgeons in Central or South America (Fig. 2, Table 3). Ninety-one percent (*n* = 127) of the studies exclusively examined populations from HICs (Table 2). Six studies (4%) exclusively examined populations from lower income countries (UMIC, LMIC, or LIC), whereas five studies (4%) evaluated populations from at least one HIC and one lower income country (Table 2). The country origins of the population in one study (1%) could not be determined [15]. Populations from HICs were represented in 95.0% of the studies (*n* = 132). Of the 26 countries represented, half (*n* = 13) were within the top 25% countries in the world for GGGI, and 73% (*n *= 19) fell within the top 50% of the 153 countries ranked by the index. One hundred and twenty-five (90%) studies exclusively examined populations from the top 50% of all GGGI ranked countries. Of the lower 50% of all countries rated by the GGGI, only 9% (*n* = 7) have study populations included in the current literature (Fig. 2, Table 4). Two countries, Japan, and Saudi Arabia were high-income economies with GGGI rankings in the bottom 50% of countries. One country, Rwanda, was a LIC ranked in the top 10 of GGGI ranked countries.

**Table 3:** Countries with study populations examined in the scoping review by continent, number of studies and World Bank income level

Continent	Studies per continent, *n* (%)	Country*	World Bank income level	Studies per country, *n* (%)
Africa	8 (4.8)	Egypt	Lower middle income	1 (0.6)
		Nigeria	Lower middle income	3 (1.8)
		Rwanda	Low income	1 (0.6)
		South Africa	Upper middle income	2 (1.2)
		Zimbabwe	Lower middle income	1 (0.6)
Asia	13 (7.9)	China	Upper middle income	1 (0.6)
		Israel	High income	1 (0.6)
		Hong Kong^†^ (SAR China)	High income	3 (1.8)
		Japan	High income	5 (3.0)
		Jordan	Upper middle income	1 (0.6)
		Saudi Arabia	High income	1 (0.6)
		Turkey	Upper middle income	1 (0.6)
Europe	31 (18.8)	Austria	High income	1 (0.6)
		Denmark	High income	1 (0.6)
		Finland	High income	2 (1.2)
		Germany	High income	2 (1.2)
		Ireland	High income	3 (1.8)
		Italy	High income	1 (0.6)
		Norway	High income	1 (0.6)
		Poland	High income	1 (0.6)
		Sweden	High income	2 (1.2)
		Switzerland	High income	3 (1.8)
		United Kingdom	High income	14 (8.5)
North America	103 (62.4)	Canada	High income	14 (8.5)
		United States	High income	89 (53.9)
Oceania	10 (6.1)	Australia	High income	6 (3.6)
		New Zealand	High income	4 (2.4)
South America	0			

* Six studies examined additional countries but did not analyze the differences between country populations [15–20]

^†^For the purposes of this review, Hong Kong (SAR, China) was treated as an entity distinct from China as the experiences of female surgeons between Hong Kong (SAR, China) and mainland China likely differ

**Table 4:** Global gender inequality index ranking of the countries with study populations included in the review

Study populations by country	Global gender gap index ranking 2020*	Economic participation and opportunity	Educational attainment	Health and survival	Political empowerment
Norway	2	11	31	95	2
Finland	3	18	1	56	5
Sweden	4	16	59	117	9
New Zealand	6	27	1	109	13
Ireland	7	43	47	113	11
Rwanda	9	79	114	90	4
Germany	10	48	103	86	12
Denmark	14	41	1	101	17
South Africa	17	92	67	1	10
Switzerland	18	34	77	110	19
Canada	19	30	1	105	25
United Kingdom	21	58	38	112	20
Austria	34	86	1	82	30
Poland	40	57	58	1	49
Australia	44	49	1	104	57
Zimbabwe	47	45	98	1	54
United States	53	26	34	70	86
Israel	64	67	1	97	64
Italy	76	117	55	118	44
China^†^	106	91	100	153	95
Japan	121	115	91	40	144
Nigeria	128	38	145	135	146
Turkey	130	136	113	64	109
Egypt	134	140	102	85	103
Jordan	138	145	81	103	113
Saudi Arabia	146	148	92	139	136

* 153 total reported countries

^†^Hong Kong (SAR, China) is not individually ranked in the GGGI index, which focuses on China as a whole

## Careers challenges

Eighty-nine percent of articles (42 of 47 articles) focusing on career challenges studied only populations from HICs (Tables 2 and 3). Three articles (7%) studied populations from HICs, UMICs, and LMICs, while two articles (4%) studied only populations from LMICs (Tables 2 and 3). Forty-two (89%) of these 47 studies exclusively examined women from the top 50% of GGGI rated countries (Tables 2, 3 and 4). Female surgeons from different countries had different perceptions of their career barriers. US surgeons attributed their career barriers to ineffective mentorship, gender stereotypes, unclear expectations, a perceived lack of belonging, and sexism in the workplace [21, 22]. Barriers to career success in Europe were ineffective mentorship, gender stereotypes, a lack of part-time career availability, and work–family conflicts [23, 24]. In Nigeria, female surgeons listed limited time with family, workload, physical effort, a lack of women in surgery, and a lack of role models as deterrents from surgical careers [25].

Two studies recommend steps to increase women in surgery. Kass et al. reported the most important factors for academic success by US female surgeons was the pursuit of mentorship (60% of respondents), setting career goals (50% of respondents) and honing writing skills and publishing (50% of respondents) [26]. To achieve better gender balance in surgery, female and male surgeons in Zimbabwe recommended better working conditions, increasing female interest in surgery, increasing the number of female role models, and changing cultural/religious beliefs [27].

## Residency and training

Thirty-seven studies focused on female surgeons in residency and training, with 86% (*n* = 32) of these articles exclusively describing HIC populations (Tables 2 and 3). Thirty-three (89%) of the articles this category focused only on the upper half of all GGGI rated countries (Tables 2 and 4). Two articles studied UMICs exclusively (South Africa by Umoetok et al.[28] and Turkey by Eyigor et al. [29]) and one article focused on a LIC, Rwanda [30]. Two studies examined populations from multiple income levels [15, 18].

### Gender-based discrimination

Fifty-one percent (*n* = 19) of the articles reviewing residency and training, highlighted female surgical trainees’ challenges with gender-based discrimination [28–46]. Gender-based discrimination was described as negative stereotyping, exclusion from networking, and physical, emotional and sexual harassment. Male colleagues were the perpetrators of 98% of reported harassment by female surgical residents in the US and 72% of these cases were from attending physicians [41]. In Canada, 25% of female medical students reported gender-based discrimination during their surgical clerkship, versus 3% of men; this discrimination was from surgeons (35%), surgical residents (25%), and nurses (17%) [33]. In the UK, 15% of female medical students were told by senior healthcare professionals that women should not be surgeons and 34% witnessed negative comments made about women as surgeons [44]. In Australia and New Zealand, the attrition of female surgical trainees was caused in part by bullying, sexual harassment, sexism, fear of repercussion, poor mental health, and a lack of support pathways [46]. In South Africa, an UMIC, 34% of female surgeons experienced physical threats, 40% experienced emotional threats, and 50% reported bullying [28]. Female surgical trainees in Turkey (an UMIC) were more likely to report gender-based discrimination if they were training in departments without female faculty (p < 0.006) [29]. Discrimination against female surgical trainees in Turkey was perpetrated by their seniors (68%), colleagues (25%), patients (6%) and hospital staff (1%) [29].

### Gender differences in surgical skill

Three studies compared the surgical skills of male and female trainees in six HICs [47–49]. Two studies examining technical capabilities in bowel anastomoses and physical strength found no significant difference in male and female surgical residents’ capabilities [47, 48]. In Rwanda, 66.7% of male and 50% of female surgeons believed that women were physically and mentally weaker than men and therefore less able to perform surgeries [30]. One female surgeon reported that there was a biological basis for the gender disparity in surgery, stating that the difference was “testosterone. Men do not fear and female do fear” [30].

### Mentorship

The impact and lack of mentorship in training were discussed in six articles from HICs [32, 36, 46, 50–52], one article from an UMIC (South Africa) [28], and one article from a LIC (Rwanda) [30]. One study from the US found that a significantly higher proportion of female medical students pursued surgery when their school had more female surgical role models (*p* < 0.0001) [50]. However, a qualitative survey in the US reported that 44% of female general surgery residents felt they lacked mentorship and that more mentorship for female surgeons is needed [36]. Similarly, in Canada, 80% of the female members of the Royal College of Physicians and Surgeons reported needing a female mentor [32]. The absence of interactions with other women in surgery was a noted reason why female trainees left surgical training in Australia and New Zealand [46]. Female surgeons in Japan had 3.6 mentors each on average, with 2.8 being male and 0.8 being female [52]. In South Africa, 75% of the female surgeons reported having a mentor, with 33.3% of their mentors being female [28]. In 22% (*n* = 7) of cases, respondents believed that the gender of their mentor made a difference in their training quality [28]. Rwanda had two female surgeons in the country as of 2018; role models for female surgical trainees in Rwanda were male surgeons and female peers [30].

## Family and work–life balance

Thirty-six studies focused on family and work–life balance with 34 articles (94%) exclusively evaluating populations from HICs. Of the 34 articles with GGGI ranked populations, 29 articles (85%) solely studied populations from the upper half of all GGGI rated countries (Tables 2, 3, and 4). One study (3%) by Abolarinwa et al. exclusively studied Nigeria, a LMIC [53]. Another study evaluated HICs, UMICs (China and South Africa) and a LMIC (Nigeria) [19].

### Pregnancy

Nineteen studies reported on the pregnancies of female surgeons [19, 53–70]. In the US, 27.5% of female surgeons had children during residency, compared with 62.4% after residency [70]. In Canada, 29.4% of female surgeons had children during residency, 7.7% prior to residency, and 55.2% after residency [62]. Female surgeons in the US who were pregnant during training reported feeling poorly judged (73.1%), pressured to schedule their pregnancies around training (55.1%), and that their work schedule negatively impacted their or their child’s health (63.3%) [65]. US female surgical trainees without children reported sadness when thinking about children (*p* = 0.047) and worry that they will never have children compared to male trainees (*p* < 0.0001) [67]. In contrast, female surgeons in Nigeria who had children gave birth more often during training (78.8%); 37.5% felt their pregnancy negatively impacted their training by increasing training time, straining relationships with instructors, or creating difficulty with scheduling outside rotations [53].

### Maternity leave

Ten studies evaluated access to childcare and maternity leave policies for female surgeons from only HICs [54, 55, 57, 61–63, 66, 69–71]. A study by Walsh et al. included populations from the US, Canada, the UK, China, Sweden, Australia, Nigeria, and South Africa [19]. In this study, Chinese female surgeons were the least likely to reduce their workload while pregnant [19]. All Nigerian female surgeons reported their spouses could not receive paid paternity leave and 86% reported that their spouses were unlikely to get unpaid paternity leave [19].

### Childcare and housework

Nine studies exclusively from HICs [57, 64, 70–76] found that women had a higher proportion of household and childcare responsibilities. Female surgeons from the US reported one to ten more hours of housework per week versus male surgeons [72]. In Germany, female surgeons spent 7.4% of their week running the household compared to 5.9% for male surgeons [70]. Female surgeons from Canada reported more hours of childcare per week compared to male surgeons (*p* < 0.0003) [74]. Twenty-seven percent of female surgeons in Switzerland completed all housework themselves [75]. In Hong Kong, more female surgeons reported having less time to rest than male surgeons (*p* = 0.038) [71]. Japanese female surgeons were more likely to report sacrificing career success or advancement for childbearing (*p* < 0.01); they had less family support for their careers than female surgeons from other countries (*p* < 0.01) [76]. Japanese female surgeons also had the least amount of personal time [76]. In Hong Kong, female surgeons reported less time for community participation and rest compared to male counterparts [71].

## Health and other topics

Nineteen studies, all from HICs and the upper half of GGGI countries, focused on other topics: interpersonal interactions (*n* = 3), payment (*n* = 8), physical health (*n* = 5), demographics (*n* = 2), and international volunteerism (*n* = 1) (Fig. 1, Table 2). Female surgeons in Poland had shorter life expectancies than the general female population (77.5 vs 86.6 years) [77]. Norwegian female surgeons drank large quantities of alcohol more frequently than non-surgeon female physicians (18% vs. 7.6%) [78]. Compared to the general population in the US, breast cancer prevalence was significantly greater in female orthopedic surgeons (*p* < 0.001) [79]. US female surgeons were more likely to receive treatment for issues relating to their hands than males (*p* = 0.028), citing instrument design (84%) and operating room table height (44%) as the cause of their symptoms [80]. In the US, female surgeons earned over $60,000 less per year than male surgeons after controlling for work hours, case volume, years in practice, practice setting and specialty (p < 0.001) [81].

## Discussion

To the author’s knowledge, this study reflects the only scoping review evaluating the experiences of female surgeons worldwide. The demographics of included studies alone provide unique insights into the literature on women in surgery. The majority of research on female surgeons was published in the past five years and focuses on women from the US or other HICs and high GGGI ranked countries. With only 26 countries in this review, we have demonstrated a large shortage of literature on female surgeons experiences compared to the reported 53 countries where female surgeons exist [19]. In particular, no literature on female surgeons was available from Central and South America, despite evidence of women working as surgeons in this region [82]. More importantly, this review has demonstrated that differences in culture, economic and educational opportunity, gender equity and women’s empowerment affect the experiences of both female surgical trainees and current female surgeons [3, 18, 83].

The first step in training and retaining more women in surgery is to support the current cohort of female surgeons worldwide, as female surgeons in North America, Europe, Oceania, Asia, and Africa identified lack of mentorship, particularly female mentorship, as a barrier to career advancement and a reason for attrition in surgical training [23, 27, 28, 30, 32, 36, 46, 52, 75]. One possible solution for this barrier is to increase the mentorship and visibility of women in surgical specialties, which has been demonstrated in the US to positively influence young women to enter surgical specialties [50]. Increasing the number of female surgeons through mentorship is less feasible in some countries. Despite evidence that women and men have equivalent physical strength and skills, the limited number of female surgeons currently in countries like Rwanda, along with the societal belief that women are less suited for the demands of surgery, limits the availability of mentors for new female surgeons [30, 47–49].

A country’s income and GGGI status can help frame the need to support their women in surgery. Rwanda is a LIC with a high ranking for global gender equality but very low ranking for educational attainment; negative attitudes towards female surgeons may stem from a deeper sociological mindset towards the educational achievements and career choices of women. Zimbabwe has a moderate GGGI ranking overall but a low ranking in educational attainment; there, both male and female surgeons believe that cultural and religious attitudes need to change in order to achieve gender equity in surgery [27]. In low-and-middle income countries with lower GGGI educational attainment rankings, working to change cultural attitudes about female education and stereotypical gender roles may be the first step towards increasing the prevalence of women in surgery.

Regardless of country income level, lower GGGI rankings can predict restrictive gender norms that limit female attainment in surgery. Populations from East Asia (Japan, Hong Kong, and China) had higher incomes (HIC and UMIC) and GGGI rankings in the lower 50%, particularly in economic participation. This dichotomy may highlight cultural structures less inclusive of female advancement. Unlike female surgeons from western countries, Japanese female surgeons reported less familial support for their careers and less leisure time. Seen as the responsibility primarily of women in countries with lower GGGI rankings and low female economic participation, domestic duties are in direct conflict with medical systems that rewards long hours and increased overtime work [76]. Therefore, the medical fields in countries with low GGGI rankings, regardless of income status, may be designed to favor the male workforce. Gender norms in these countries further strain female surgeons’ work–life balance and career attainment. Future initiatives in these countries should target cultural attitudes about women’s domestic roles and economic participation along with policies to increase flexible work schedules for female surgeons.

In HICs with high GGGI rankings, geographic and cultural differences affect surgeons’ perceptions and barriers. Female surgeons did more household work than male counterparts. Child-related barriers were reported more by Europeans than Americans [21–24], which was surprising given the abundance of state and hospital sponsored childcare in Europe [84]. The ubiquity of childcare in Europe may have created an environment where small gaps in childcare services are a perceived barrier, while childcare in the US is completely privatized.

Countries with extended family support systems do not face the same childcare challenges. Nigeria has lower income and low GGGI, but most Nigerian female surgeons were able to have children during residency without barriers (79%), unlike women in the US and UK (28% and 47%, respectively) [53, 61, 70]. With older relatives living in the home, Nigerian women can rely on an extended family system to run households [53, 85]. This extended family system is common in countries with similar cultural norms, allowing female surgeons from lower income and lower GGGI countries to achieve greater work–life balance at earlier stages of their careers.

Discrimination against female surgeons during their training, career, and pregnancy, was a common finding in high GGGI and higher income countries (HICs, UMICs) countries such as the US, UK and South Africa [28, 31–42, 65]. Discrimination and harassment were perpetuated most commonly by male colleagues in positions of power, which increases work-related stress and burnout while decreasing retention rates among female surgeons [33, 41]. High GGGI ranked countries may have more awareness towards discrimination against professional women. In lower ranked GGGI countries, the lack of studies on gender-based discrimination against female surgeons underrepresents the extent of the problem. A lack of awareness or minimal consequences for discrimination in low GGGI countries contributes to the absence of advocacy against discrimination. In a Turkish example, increasing the number of female surgeons in leadership is one way to reduced gender-based discrimination [29]; this model could be replicated in similar environments.

Female surgeons in HICs and high GGGI countries reported worse health outcomes compared to male surgeons and the general population. Studies from HICs reported that female surgeons had higher rates of cancer, alcohol consumption, and musculoskeletal ailment accompanied by lower life expectancies across European and North American countries [77–80]. As all the literature on female surgeons’ health focused on HICs, this finding could not be compared to female surgeons in lower income countries. But, the difference between female surgeons and the general population may be less obvious in environments where average health and lifespan standards are lower [86]. It is also possible that a career as a surgeon may provide a higher standard of living in lower income countries, which can counteract some of the health detriments from the profession seen in HICs. However, further studies would be needed to validate these hypotheses.

This study is limited by its design as a scoping review, as such there was no formal evaluation of the quality of evidence or risk of bias in the studies. Additionally, the lack of reporting from Central and South America limits this study’s generalizability to this region. The lack of studies from South or Central America likely has to do with our inclusion and exclusion criteria, specifically with regards to literature available in English. During the review many studies on South America emerged, one discussed the proportions of female surgeons in Brazil [82], but none specifically discussed the experiences of female surgeons from any country in this region. As 91% and 90% of studies exclusively examined HICs and high GGGI countries, respectively, the role of income level and GGGI ranking in female surgeons’ experiences cannot be generalized without more diversity in the literature. The lack of reporting from lower income and lower GGGI countries limits the ability to provide definitive, context-specific recommendations to improve female surgeon experiences and participation.

### Conclusions

Different geographic regions along with cultural and societal norms influence gender equity and the experiences of women in surgery. Universally, women from all regions reported a lack of mentorship as a barrier to advancement. An overwhelming majority of studies originated in high-income, high GGGI countries in Europe and North America. In HICs, surgical trainee abilities are seen as equal between men and women, but women endure discrimination from male co-workers and reported more child-related barriers to their careers than their male counterparts. While female surgeon abilities were seen as inferior in some lower income countries, limited studies suggest that women may have more child rearing support and be less likely to delay childbearing. The effects of income and GGGI are complex, as neither independently predict gender equity in surgery. More studies in lower income and lower GGGI countries are needed to understand this relationship and how to improve the female surgical experience to increase surgical capacity worldwide.

## Supplementary information

**Additional file 1**. PRIMSA-ScR-Checklist.

**Additional file 2**. Scoping Review Protocol.

## Data Availability

Data sharing is not applicable to this article as no datasets were generated or analyzed during the current study.

## Abbreviations

GGGI: Global gender gap index
US: United States of America
UK: United Kingdom
HIC: High-income country
UMIC: Upper-middle income country
LMIC: Lower-middle income country
LIC: Low-income country
