# Supplementary material for: The experiences of female surgeons around the world: a scoping review
Source: Hum Resour Health. 2020 Oct 28;18:80. doi: 10.1186/s12960-020-00526-3 (PMC7594298; doi:10.1186/s12960-020-00526-3)
Supplement: Supplementary file 2 — Additional file 2. Scoping Review Protocol. [file 12960_2020_526_MOESM2_ESM.docx]

The Experiences of Female Surgeons Around the World: A Scoping Review Protocol

Meredith D. Xepoleas, Naikhoba C.O. Munabi, Allyn Auslander, William P. Magee III & Caroline A. Yao

**Background**

Women now representing 50% of current medical school matriculants in the U.S. (1). This shift is not reflected in surgical specialties, where men represent 61.6% of U.S. and 73% of U.K. practicing surgeons (2, 3). Five-billion people currently lack access to safe, affordable surgical care globally and many countries need an increase in surgical providers to reach the recommended 20 per 100,000 population; concurrently, the number of female surgeons in low- and middle-income countries (LMICs) rose disproportionately slower than female representation in other medical specialties (4-6). With the majority of LMICs struggling to build an adequate surgical workforce, expanding the participation of women in surgery has been proposed as one way to help alleviate the global burden of surgery (5, 6). As surgery continues to be a male-dominated field, the discrepancies in experiences between genders have not been as well documented worldwide. Primary research studies that document these experiences in the literature are narrow in both their study focus and populations. Providing a greater understanding the career experiences of women in surgery is essential to expand the female workforce, improve the professional surgical environment, and retain existing female surgeons. For the purposes of this study, the female surgical experience was defined as any difference in attitude, treatment, behavior or consequence that results from a surgeon’s female gender. This scoping review seeks to answer the questions, (1) what are the experiences of female surgeons around the world and (2) how do those experiences differ due to variations in geography, national income and cultural ideas of gender equity? To understand the global variability of national income and cultural ideas of gender equity in female surgeon experiences, we will evaluate studies according to the country region, World Bank Income Level Group, and Global Gender Gap Index (GGGI), respectively. The World Bank classifies countries into four categories according to gross national income per capita: low, lower-middle, upper-middle, and high income (7). These income level groupings indicate a country’s economic capabilities, associated resources, and opportunities that may be available to the population within. The Global Gender Gap Index is a weighted rating comprised of scores for economic participation and opportunity, educational attainment, health and survival, and political empowerment. GGGI ratings contextualizes the experiences of women around the world in a social and professional capacity. Lower scores and rankings correspond to less equality for women (8).

A preliminary search for systematic and scoping reviews examining this topic has been performed on JBI Database of Systematic Reviews and Implantation Reports and PubMed on August 22, 2019. No studies were found.

*Aim*

The female surgical experience is a very broad topic for which we aim to synthesize the current knowledge of female surgeons’ professional experiences globally and identifies where gaps in gender equity are most evident internationally.

*Objective*

The information from this review will be used to inform future training programs and recommend professional, educational and institutional initiatives and policies. We hope to inspire the development of new strategies to alleviate the burden of surgical disease and increase surgical capacity through empowering women globally.

*Scoping Review Questions*

This review seeks to explore, through the available literature, what the experiences of female surgeons are around the world. The specific review questions to be addressed are:

1. What are the experiences of female surgeons around the world?
2. How do those experiences differ due to variations in geography, national income and cultural ideas of gender equity?

**Inclusion Criteria**

*Types of participants*

This review will consider all female surgeons, female surgical residences, and female medical students that indicate they are pursing the surgical field.

*Concept*

The experiences of female surgeons that differ from male surgeons as result of the woman’s identity as both a surgeon and a female. These experiences can be any difference in attitude, treatment, behavior or consequence that results from a surgeon’s female gender.

*Context*

For the purposes of this review, the context will include every country and society in order to evaluate the variations in geography, national income and cultural ideas of gender equity that all impact the female surgical experience.

*Types of Evidence Sources*

All original studies published in peer-reviewed journals that examine the female surgical experiences will be included. This includes qualitative studies, cross-sectional analysis, questionnaires, longitudinal analysis, controlled trials, systematic reviews, and observational studies. Editorials and personal anecdotes will be excluded for their potential bias.

**Search Strategy**

The search strategy will be designed to access published materials and will comprise two stages:

(1) A limited search of PubMed to identify relevant keywords contained in the title, abstract and subject descriptors.

(2) Terms identified in this way will be used in an extensive search of the literature.

The initial search terms will be ‘female surgeons’, ‘women in surgery’, and ‘female surgical experience’. There will be no time period excluded in order to evaluate all of the literature on female surgeons. Articles published in English for the authors’ ability to assess the study will be searched in the following databases:

MEDLINE (Ovid)

PubMed

Web of Science

Full copies of articles identified by the search, and considered to meet the inclusion criteria, based on their title, abstract and subject descriptors, will be obtained for qualitative data synthesis. Two reviewers will independently select articles against the inclusion criteria. Discrepancies in reviewer selections will be resolved at a meeting between reviewers prior to selected articles being retrieved.

*Source of Evidence Selection*

After searching the relevant terms identified across each of the three databases, the results will be download into files compatible with Microsoft Excel 2010 (Microsoft Corporation). After all the results from each database are entered into the same file, duplicates will be removed through excel. Any duplicates found in later parts of the search will be removed. After deduplications, one author (M.X.) will conduct the initial review and excluded articles that did not meet inclusion criteria according to title only. Two authors (M.X. and N.M.) will review the remaining studies according to their abstracts and individually excluded articles that did not meet inclusion criteria. The remaining articles will be input into a chart created on Microsoft Excel. This chart will be used to extract the relevant study characteristics: study design, publication year, study population, country or countries of the study population, gender distribution of the study population, the category of the female surgical experience, and the study’s main findings. From this chart, the full text articles of each studies input into this chart will be individually reviewed by (M.X. and N.M.). Study characteristics input into the excel sheet will confirm relevance. Studies that do not meet the inclusion criteria will be excluded. A final list of included studies will be compiled by resolving any differences between the two authors’ lists through discussion. Once a file list has been complied, World Bank Income Level and GGGI will be searched for the countries included in the review.

**Data Extraction**

A chart has been developed to extract data from the included studies and to confirm the relevance of full text articles.

1. Year of publication
2. Title
3. World bank income level
4. Study population
5. Country of study population
6. Study Design
7. Funding Source
8. Gender distribution of the study population
9. The category of the female surgical experience
10. The study’s main findings that relate to the scoping review questions

*Analysis of Evidence*

Percentages will be used to describe the numeral data from the amount and characteristics of studies included in the review. Qualitative data from the studies will be the main source of data for the experiences of female surgeons.

*Presentation of the results*

1. A flow chart will be developed to explain the search process.
2. A map will be used to explore the number of studies per country.

**References**

1. Colleges AoAM. Table A-7.2: Applicants, First-Time Applicants, Acceptees, and Matriculants to U.S. Medical Schools by Sex, 2010-2011 through 2019-2020. 2019.
2. Skinner H, Burke JR, Young AL, Adair RA, Smith AM. Gender representation in leadership roles in UK surgical societies. Int J Surg. 2019;67:32-6.
3. DataUSA. Physicans & surgeons. - Gender Composition DataUSA: DataUSA; 2017 [cited 2019. Available from: <https://datausa.io/profile/soc/291060/>. Accessed on 11/21/19
4. Blakemore LC, Hall JM, Biermann JS. Women in surgical residency training programs. J Bone Joint Surg Am. 2003;85(12):2477-80.
5. Meara JG, Greenberg SL. The Lancet Commission on Global Surgery Global surgery 2030: Evidence and solutions for achieving health, welfare and economic development. Surgery. 2015;157(5):834-5.
6. Organization WH. Delivered by women, led by men: A gender and equity analysis of the global health and social workforce. Human Resources for Health Observer Series No 24. 2019( Licence: CC BY-NC-SA 3.0 IGO.).
7. Group TWB. World Bank Country and Lending Groups. 2019 [cited 2020. Available from: <https://datahelpdesk.worldbank.org/knowledgebase/articles/906519-world-bank-country-and-lending-groups>. Accessed on 11/20/19
8. Forum WE. The Gender Gap Report 2018 World Economic Forum2018 [Available from: <http://reports.weforum.org/global-gender-gap-report-2018/the-global-gender-gap-index-2018/>. Accessed on 11/20/19
